# A Case-Control Study on Risk Factors and Their Interactions with Prediabetes among the Elderly in Rural Communities of Yiyang City, Hunan Province

**DOI:** 10.1155/2019/1386048

**Published:** 2019-02-18

**Authors:** Zhao Hu, Fan Gao, Lulu Qin, Yang Yang, Huilan Xu

**Affiliations:** ^1^Department of Social Medicine and Health Management, Xiangya School of Public Health, Central South University, Changsha, China; ^2^Department of Health Monitoring, Xi'an Center for Disease Control and Prevention, Xi'an, Shaanxi, China; ^3^Department of Social Medicine and Health Management, School of Medicine, Hunan Normal University, Changsha, China

## Abstract

**Background:**

The prevalence of prediabetes has been increasing significantly in recent years. Individuals with prediabetes have an increased risk of developing diabetes and cardiovascular diseases. The objectives of this study were to identify risk factors for prediabetes and their interactions among the elderly in rural communities of Hunan Province and to provide a scientific basis for prediabetes prevention.

**Methods:**

A case-control study was conducted to explore risk factors for prediabetes among the elderly in rural areas. The general sociodemographic information, lifestyle behaviours, and physiological results of elderly individuals with prediabetes and controls were collected by a questionnaire and laboratory testing. Conditional logistic regression was performed to identify the risk factors for prediabetes among the elderly, and additive interactions were used to analyse the interactions between risk factors.

**Results:**

A total of 425 elderly subjects with prediabetes were included in the case group, and 425 elderly subjects with normal plasma glucose levels were included in the control group. The main risk factors for prediabetes among the elderly in rural communities of Hunan Province were a family history of diabetes (OR = 2.48; 95% CI: 1.13, 5.46), physical inactivity (OR = 3.27; 95% CI: 1.95, 5.49), a lack of health literacy on diabetes prevention and control (OR = 3.26; 95% CI: 1.62, 6.55), hypertension (OR = 2.01; 95% CI: 1.38, 2.93), overweight (OR = 2.53; 95% CI: 1.67, 3.81), obesity (OR = 3.08; 95% CI: 1.48, 6.40), and a high waist-to-hip ratio (WHR) (OR = 2.26; 95% CI: 1.45, 3.51). Additive interactions for prediabetes were detected between a high WHR and physical inactivity, with a relative excess risk due to interaction (RERI) of 6.30 (95% CI: 0.42, 12.18), and between a high WHR and overweight or obesity, with an RERI of 2.92 (95% CI: 0.56, 5.29).

**Conclusion:**

The independent risk factors for prediabetes are a family history of diabetes, physical inactivity, a lack of health literacy on diabetes prevention and control, hypertension, overweight or obesity, and a high WHR. A high WHR has additive interactions with physical inactivity and overweight or obesity for the risk of prediabetes. These findings have significant implications for prediabetes prevention among the elderly in rural areas.

## 1. Introduction

Prediabetes is defined as an intermediate state characterized by glycaemic parameters above normal levels but below the diabetic threshold, including impaired fasting glucose (IFG) and impaired glucose tolerance (IGT) [1, 2]. Many studies have indicated that impaired glucose regulation is already present among the prediabetic population. As discussed by Butler et al. [3], obese humans with IFG had a 40% deficit in the relative beta-cell volume compared with nondiabetic obese subjects. Moreover, without timely and effective interventions, prediabetes is very likely to progress to diabetes within a few years. As demonstrated by Nichols et al. [4], approximately 1.34% of newly diagnosed and 5.56% of previously diagnosed IFG patients developed diabetes within a year, and the average times for the development of diabetes were 41.4 months and 29.0 months, respectively. Similar to Nichols et al.'s findings, Rasmussen et al. [5] found that progression rates from IFG and IGT to diabetes were 11.8 and 17.0 per 100 person-years over 3.5 years, and they were particularly high in the first year. In addition, a diabetes prevention prospective study conducted in China [6] discovered that the cumulative incidence of diabetes was greater than 90% among prediabetic subjects in the nonintervention group.

Previous studies have demonstrated that subjects with prediabetes have an increased risk of not only diabetes but also cardiovascular disease [7, 8]. Moreover, several meta-analyses have indicated that prediabetes was associated with a greater than 10% increased risk of all-cause mortality [9, 10]. Some studies have indicated that the free fatty acids and insulin resistance associated with prediabetes provoke molecular mechanisms that alter the function and structure of blood vessels, trigger vasoconstriction and inflammation, and promote coronary atherosclerosis [7, 11]. The incidence of diabetes is closely related to various risk factors, including a family history of diabetes, advanced age, obesity, hypertension, and physical inactivity [12–15]. However, several studies have also shown that many identical risk factors for diabetes are already present in prediabetic individuals [16–20].

According to a survey conducted in China in 2010, the prevalence of prediabetes was estimated to be greater than 57% among the elderly, which is higher than that of the general population [19]. Moreover, rural residents had a slightly higher prevalence of prediabetes than urban residents. In addition, the incidence of diabetes among the elderly with prediabetes is already high in rural areas [20]. However, few studies have investigated the risk factors for prediabetes among rural residents in China. Therefore, the aim of our current study was to investigate risk factors for prediabetes and their additive interactions among the elderly in rural areas of Hunan Province. We hope that this study can also provide a scientific basis for prediabetes prevention for elderly individuals living in rural areas in China.

## 2. Material and Methods

### 2.1. Study Design and Sample Size

This was a case-control study carried out in rural areas of Yiyang City of Hunan Province in China between April and July 2015. This study was registered at the Chinese Clinical Trial Registry (trial registration number: ChiCTR-IOR-15007033). All participants signed the respective consent forms. According to the sample size estimation formula for a 1 : 1 paired case-control study and a previous study in China [21], a total of 403 participants were needed in each group.

### 2.2. Study Population and Procedures

In this study, a multistage cluster random sampling method was used to select a representative sample of the rural population with prediabetes. In the first stage, 2 of 7 counties were randomly selected from Yiyang City in Hunan Province, namely, Yuanjiang and Nanxian. In the second stage, 2 of 11 townships in Yuanjiang and 2 of 9 townships in Nanxian were randomly selected. In the third stage, 25% of rural villages were randomly selected from each selected township (each township contains 30-50 villages). In the final stage, all households with elderly individuals in each selected village were identified through oral glucose tolerance test (OGTT) results.

### 2.3. Case and Control Definitions

The elderly with prediabetes determined by the OGTT constituted the cases [22], which included three types of subjects: (1) IFG (fasting plasma glucose of 110–126 mg/dL and a 2-hour postglucose load of <140 mg/dL), (2) IGT (fasting plasma glucose ≤ 110 mg/dL and a 2-hour postglucose load of 140-200 mg/dL), and (3) IFG+IGT. Elderly individuals with diabetes and other serious physical or mental illness were excluded. Elderly subjects with normal glucose tolerance were matched with prediabetic subjects of the same gender and similar age (within 3 years) living in the same village.

### 2.4. Data Collection and Measurements

The sociodemographic information of the participants was collected by professional trained investigators through a face-to-face interview using a set of structured questionnaires, which included age, gender, occupation, marital status, education, personal annual income, the presence of other chronic diseases, family history of diabetes, and history of hyperglycaemia. Occupation was classified into one of four categories: farmer, worker, retired, and other occupation, and other occupations included individual household and unemployed. Marital status was classified as married and nonmarried, with nonmarried status including never married, divorced, widowed, and living with a partner without a marriage certificate. Education was assessed by asking the participants to select their highest level of education completed from the following choices: less than or equal to 6 years and more than 6 years. Personal annual income was classified as ≤2800 CNY/year and >2800 CNY/year. A family history of diabetes was defined when first-degree relatives were diabetic.

Lifestyle behavioural information including dietary patterns, physical activity, smoking status, alcohol intake, sleep duration, history of steroid drug use, and health literacy on diabetes prevention and control was collected. Dietary patterns were assessed using the China Health and Nutrition Survey (CHNS) questionnaire [23], which covered food consumption over the last 12 months, and the Diet Balance Index (DBI-07) was calculated to evaluate dietary quality [24]. The International Physical Activity Questionnaire- (IPAQ-) long version was used to investigate the physical activity levels of the participants in the last week in four domains, with a minimum duration of 10 min per session, which were classified into three levels: low, moderate, and high. The Questionnaire of Health Literacy of Diabetes Mellitus of the Public (HLDMP) designed by the Chinese Center for Health Education was used to assess the health literacy of individuals regarding diabetes prevention and control. Smoking was defined as continuous smoking for more than 6 months and smoking at least 1 cigarette per day [25]. Alcohol intake was defined as drinking one glass of wine (approximately 250 mL of beer, 100 mL of sake, or 20 mL of liquor) [26]. Sleep duration was classified as less than 6 hours, 6-8 hours, and above 8 hours [27]. Participants with a DBI score of zero or lower than 20% of the total scores were defined as having a balanced diet [24]. Physical inactivity was defined as a low physical activity level according to the criteria of the IPAQ [28]. An HLDMP score ≥ 32.4 was defined as having health literacy on diabetes prevention and control [29].

Anthropometric measurements included height, weight, blood pressure, waist circumference, and hip circumference. Height was measured to the nearest 0.1 cm using a stadiometer, and weight was measured without shoes and in light indoor clothing to the nearest 0.1 kg. Blood pressure was measured twice using an electronic blood pressure monitor (LifeSource UA-767PV, A&D Medical, San Jose, California, USA), and the means were used in the analyses. The body mass index (BMI) was calculated with the following formula: BMI = kg/m^2^; the cut-off values for the Chinese population for normal weight, overweight, and obesity are 18.5 kg/m^2^, 24.0 kg/m^2^, and 28.0 kg/m^2^, respectively [30]. The waist-to-hip ratio (WHR) was calculated by dividing the waist circumference by the hip circumference. A WHR > 0.9 in men and >0.8 in women was defined as a high WHR [31]. Hypertension was defined as a systolic blood pressure ≥ 140 mmHg and/or a diastolic pressure ≥ 90 mmHg [32]. The plasma glucose level was measured using the hexokinase enzymatic method, and the serum lipid level was measured enzymatically with commercially available reagents in the biochemical laboratory of the primary care center in each village. Individuals with dyslipidaemia were identified according to the 2016 Chinese guideline for the management of dyslipidaemia in adults [33].

### 2.5. Statistical Analysis

The data were analysed using SPSS version 18.0 (SPSS/IBM, Armonk, New York, USA) and SAS version 9.2 (SAS Institute Inc., Cary, NC, USA). Continuous variables are presented as the mean ± SD, while categorical variables are presented as numbers and percentages. A matched chi-square test was used to analyse differences in sociodemographic characteristics, lifestyle behaviours, and physiological features between the case group and the control group. Binary multivariate conditional logistic regression was used to analyse the risk factors for prediabetes, and the Excel table prepared by Andersson et al. was used to calculate additive interactions between the risk factors [34]. Three measures are used to test additive interactions: the relative excess risk due to interaction (RERI), the attributable proportion due to interaction (AP), and the synergy index (S). If the RERI and AP are equal to 0 or S is equal to 1, no additive interaction exists. All statistical tests were two-tailed, and *p* < 0.05 was considered statistically significant.

## 3. Results

### 3.1. The Characteristics of the Study Population

A total of 2144 elderly individuals in rural areas of Yiyang underwent the OGTT for prediabetes screening, and 461 elderly individuals with prediabetes were screened out in this population. Among the 461 prediabetic subjects, 425 subjects completed the questionnaire regarding risk factors for prediabetes (30 did not respond and 6 had incomplete data; response rate: 93.5%). Then, 425 subjects with normal glucose tolerance were matched with the 425 prediabetic individuals of the same gender and similar age (within 3 years) living in the same village. The average age was 69.4 ± 6.5 years in the case group and 69.5 ± 6.6 years in the control group. The male-to-female ratio was 1 : 1.36 in the case and control groups.

### 3.2. Univariate Analysis of Risk Factors for Prediabetes

#### 3.2.1. Sociodemographic Characteristics

The matched chi-square test showed that the difference in the distribution of a family history of diabetes between the two groups was statistically significant (*p* = 0.001), indicating that the elderly subjects with a family history of diabetes were at a higher risk of prediabetes. No statistically significant differences were found in the distributions of age, occupation, marital status, education, personal annual income, and history of hyperglycaemia between the two groups (all *p* > 0.05). The results are shown in Table 1.

#### 3.2.2. Risk Factors Related to Lifestyle Behaviours for Prediabetes

The matched chi-square test results showed statistically significant differences in the distributions of physical activity and health literacy on diabetes prevention and control between the two groups (all *p* < 0.001), indicating that the elderly subjects with inadequate physical activity or health literacy were at a higher risk for the development of prediabetes. No statistically significant differences in dietary quality, sleep duration, smoking, alcohol intake, and history of steroid drug use were found between the two groups (all *p* > 0.05). The results are shown in Table 2.

#### 3.2.3. Risk Factors Related to Physiological Features and Other Chronic Diseases for Prediabetes

The prevalence of hypertension and dyslipidaemia was statistically different between the two groups (all *p* < 0.01). The results also showed statistically significant differences in the distributions of the BMI and the WHR between the two groups (all *p* < 0.001), indicating that the elderly subjects who were overweight and obese or had a high WHR were at a higher risk of developing prediabetes. The results are shown in Table 3.

### 3.3. Multivariate Analysis of Risk Factors for Prediabetes

Binary multivariate conditional logistic regression showed that the independent risk factors for prediabetes among the elderly were a family history of diabetes (OR = 2.48; 95% CI: 1.13, 5.46), physical inactivity (OR = 3.27; 95% CI: 1.95, 5.49), a lack of health literacy on diabetes prevention and control (OR = 3.26; 95% CI: 1.62, 6.55), hypertension (OR = 2.01; 95% CI: 1.38, 2.93), overweight (OR = 2.53; 95% CI: 1.67, 3.81), obesity (OR = 3.08; 95% CI: 1.48, 6.40), and a high waist-to-hip ratio (OR = 2.26; 95% CI: 1.45, 3.51) after controlling for other variables, including age, occupation, marital status, education, personal annual income, history of hyperglycaemia, dietary patterns, sleep duration, smoking, alcohol intake, history of steroid drug use, dyslipidaemia, and coronary heart disease. The results are shown in Table 4.

### 3.4. Interactions of Risk Factors with Prediabetes

In this study, multivariate conditional logistic regression was used to calculate the regression coefficients and covariance matrix after adjustment, and the adjusted factors included all the factors mentioned above except for two factors for which the interaction between them was examined. Microsoft Excel was used to calculate the RERI, AP, and S, and their 95% CIs were based on the results from the logistic regression. Additive interactions were detected between a high WHR and physical inactivity, with an RERI of 6.30 (95% CI: 0.42, 12.18), AP of 0.70 (95% CI: 0.50, 0.91), and S of 4.84 (95% CI: 1.65, 17.85). Additive interactions were also detected between a high WHR and overweight or obesity, with an RERI of 2.92 (95% CI: 0.56, 5.29), AP of 0.54 (95% CI: 0.28, 0.81), and S of 3.02 (95% CI: 1.17, 7.46). The results are shown in Table 5.

## 4. Discussion

Our study is the first to compare sociodemographic characteristics, lifestyle behaviour factors, and physiological features between prediabetic subjects and subjects with normal glucose tolerance among the elderly in rural communities of China. In this study, we found that a family history of diabetes, physical inactivity, a lack of health literacy on diabetes prevention and control, hypertension, overweight, obesity, and a high WHR were the risk factors for prediabetes after adjustments for other covariates. Further, we also found that a high WHR had additive interactions with physical inactivity and overweight or obesity. In other words, compared to the presence of a high WHR alone, the combined presence of a high WHR, physical inactivity, and overweight or obesity can greatly increase the possibility of developing prediabetes.

Epidemiological studies have shown that a family history of diabetes was an important risk factor for diabetes, and previous studies have also shown that this factor remained significantly associated with the prevalence of prediabetes [19, 35, 36], which is consistent with the results of our study. With regard to lifestyle behaviour risk factors, previous studies have indicated that smoking, alcohol consumption, and physical inactivity can increase the risk for the development of prediabetes [37–39], which is similar to the results of our study. For smoking, the nicotine in tobacco can raise blood sugar levels by increasing the levels of norepinephrine, epinephrine, glucocorticoid, and growth hormone glucagon levels in the body and can also reduce insulin secretion, thus causing insulin resistance [40, 41]. Alcohol consumption can also interfere with glucose metabolism in the body, thus increasing blood glucose levels. However, studies hold different views on the effect of physical activity on the development of prediabetes. Some studies have demonstrated that adequate physical activity can help maintain the weight balance and improve the distribution of central fat, which in turn helps promote lipid and glucose metabolism [39, 42]. However, Okwechime et al. [43] showed that the level and intensity of physical activity were not significantly related to the prevalence of prediabetes among adults aged 18 years or older in Florida. For health literacy, many studies have indicated that people with inadequate health literacy were more likely to have poor glycaemic control and were less likely to achieve tight glycaemic control than people with adequate health literacy [44, 45]. However, another study indicated that diabetes-specific health literacy was significantly and positively associated with self-care practice acumen but was not independently associated with the HbA1c level [46]. Thus, the association between health literacy and diabetes outcomes represents a direction for future research. For physiological features, several studies have demonstrated that hypertension, dyslipidaemia, and coronary heart disease were independent risk factors for the development of prediabetes [39, 47, 48]. Previous studies have shown that hypertension, dyslipidaemia, and coronary heart disease frequently occur together with diabetes in an individual, indicating that a significant association exists between these diseases and glucose metabolism [13, 49, 50]. The findings regarding the association between obesity and prediabetes are consistent with previous findings where BMI and WHR increases were found to be associated with an increased risk of prediabetes [51, 52]. The mechanisms by which obesity contributes to abnormal glucose metabolism appear to involve the endocrine activity of adipose tissue, and the abdominal adipose cells secrete several cytokines and adipokines that can have deleterious cardiometabolic effects, including effects on glucose control [53].

Consistent with the findings for the interactions of risk factors for prediabetes in the present study, Hilding et al. [54] indicated an interaction between a family history of diabetes and obesity for prediabetes in women. In addition, a positive interaction between overweight or obesity and triglycerides for abnormal glucose regulation was found by Zhao et al. [47], and an additive interaction between poor sleep quality and short sleep duration for IFG was found by Lou et al. [55]. Our study outcomes extend the findings of previous research, indicating an additive interaction between obesity and physical inactivity for prediabetes in the elderly. Some studies have demonstrated that physical inactivity slightly increased the risk of obesity [56, 57]. In addition, obesity may lead to physical inactivity [58]. These results show that physical inactivity and obesity will reinforce each other, which may explain the interaction between obesity and physical inactivity for prediabetes to some extent. Therefore, promoting physical activity and weight loss may represent a feasible strategy for prediabetes prevention.

Regarding the design of our study, we effectively controlled the selection bias through the matching criteria for the cases and controls, which included matching according to the same village, the same gender, and a similar age (within 3 years). With regard to the research tools, the IPAQ, CNHS, and HLDMP had high reliability and validity and have been used in previous studies. Although Zhao et al. [47] previously reported the risk factors for prediabetes and their interactions among rural residents in China, our study explored many risk factors for prediabetes that they did not include, such as a family history of diabetes and health literacy. Moreover, our study found an additive interaction between a high WHR and physical inactivity, which emphasizes the necessity of increasing physical exercise among the elderly with a high WHR. However, our study has also several limitations. First, this is an observational research study, and causation cannot be inferred. Second, all information was collected via face-to-face interviews; therefore, an interviewer bias is unavoidable to some degree, but we minimized this bias by training the interviewers and controlling the quality. Third, our study is a 1 : 1 paired case-control study, and many risk factors that are associated with prediabetes, such as a history of hyperglycaemia and a family history of diabetes, may be very rare in the control group. In future statistical analyses, these factors will not be included in the regression equation and may be excluded as risk factors after logistic regression, reflecting a phenomenon defined as “complete separation” by Hosmer and Lemeshow [59]. Finally, the development of disease is influenced by genetic factors and environmental factors, and a previous study has already reported an additive interaction between a family history of diabetes and obesity for the risk of prediabetes [54]. However, interactions between a family history of diabetes and other risk factors for prediabetes were not found in our study, possibly because the sample size was relatively small. Therefore, further studies with larger, more representative samples are needed to confirm these findings.

## 5. Conclusion

The independent risk factors for prediabetes are a family history of diabetes, physical inactivity, a lack of health literacy on diabetes prevention and control, hypertension, overweight, obesity, and a high WHR among the elderly in rural communities of China. A high WHR shows additive interactions with physical inactivity and overweight/obesity for prediabetes. The findings have significant implications for the prevention of prediabetes in rural areas.

## Figures and Tables

**Table 1:** Sociodemographic characteristics of the study subjects.

Variables	Cases (*n* = 425)		Controls (*n* = 425)		Value *χ*^2^	*p* value
	No.	%	No.	%		
Age (years)						
60-69	232	54.6	236	55.5	4.40	0.111
70-79	169	39.8	161	37.9		
≥80	24	5.6	28	6.6		
Gender						
Male	180	42.4	180	42.4	—	1.000
Female	245	57.6	245	57.6		
Occupation						
Farmer	194	45.6	207	48.7	8.26	0.220
Worker	98	23.1	99	23.3		
Retired	69	16.2	59	13.9		
Other occupation	64	15.1	60	14.1		
Marriage status						
Married	308	72.5	303	71.3	0.10	0.756
Nonmarried	117	27.5	122	28.7		
Education						
≤6 years	344	80.9	353	83.1	0.51	0.474
>6 years	81	19.1	72	16.9		
Personal annual income						
≤2800 CNY/year	89	20.9	76	17.9	1.17	0.279
>2800 CNY/year	336	79.1	349	82.1		
Family history of diabetes						
Yes	36	8.5	13	3.1	10.30	0.001
No	389	91.5	412	96.9		
History of hyperglycaemia						
Yes	27	6.4	21	4.9	0.57	0.451
No	398	93.6	404	95.1		

**Table 2:** Lifestyle behaviour risk factors for prediabetes among the elderly.

Variables	Cases (*n* = 425)		Controls (*n* = 425)		Value *χ*^2^	*p* value
	No.	%	No.	%		
Physical inactivity						
Yes	88	20.7	34	7.5	30.36	<0.001
No	337	79.3	393	92.5		
Balanced diet						
Yes	227	53.4	254	59.8	3.47	0.062
No	198	46.6	171	40.2		
Sleep duration (hours)						
<6	55	12.9	51	12.0	0.27	0.966
6-8	269	63.3	269	63.3		
>8	101	23.8	105	24.7		
Smoking						
Yes	130	30.6	107	25.2	3.59	0.058
No	295	69.4	318	74.8		
Alcohol intake						
Yes	99	23.3	78	18.4	3.36	0.066
No	326	76.7	347	81.6		
History of steroid drug use						
Yes	17	4.0	12	2.8	0.59	0.442
No	408	96.0	413	97.2		
Health literacy on diabetes prevention and control						
Yes	13	3.1	46	10.8	17.36	<0.001
No	412	96.9	379	89.2		

**Table 3:** Physiological features and other chronic diseases among the elderly.

Variables	Cases (*n* = 425)		Controls (*n* = 425)		Value *χ*^2^	*p* value
	No.	%	No.	%		
Hypertension						
Yes	194	45.6	127	29.9	24.07	<0.001
No	231	54.4	298	70.1		
Dyslipidaemia						
Yes	171	40.2	124	29.2	11.39	0.001
No	254	59.8	301	70.8		
Coronary heart disease						
Yes	51	12.0	47	11.1	0.12	0.728
No	374	88.0	378	88.9		
BMI						
Lean	17	4.0	25	5.9	52.32	<0.001
Normal	226	53.2	316	74.3		
Overweight	128	30.1	70	16.5		
Obesity	54	12.7	14	3.3		
WHR						
High	355	83.5	300	70.6	21.93	<0.001
Normal	70	16.5	125	29.4		

BMI: body mass index; WHR: waist-to-hip ratio.

**Table 4:** Multivariate logistic analysis of the risk factors for prediabetes among the elderly.

Variables	*B*	SE	Wald	*p* value	Adjusted OR (95% CI)^†^
Family history of diabetes					
No					1.00
Yes	0.91	0.40	5.10	0.024	2.48 (1.13, 5.46)
Physical inactivity					
No					1.00
Yes	1.18	0.27	20.01	<0.001	3.27 (1.95, 5.49)
Health literacy on diabetes prevention and control					
Yes					1.00
No	1.18	0.36	10.98	0.001	3.26 (1.62, 6.55)
Hypertension					
No					1.00
Yes	0.70	0.19	13.38	<0.001	2.01 (1.38, 2.93)
BMI					
Normal					1.00
Lean	0.37	0.40	0.86	0.355	1.45 (0.66, 3.18)
Overweight	0.93	0.21	19.44	<0.001	2.53 (1.67, 3.81)
Obesity	1.12	0.37	9.08	0.003	3.08 (1.48, 6.40)
WHR					
Normal					1.00
High	082	0.23	13.11	<0.001	2.26 (1.45, 3.51)

†Adjusted for age, occupation, marital status, education, personal annual income, history of hyperglycaemia, dietary patterns, sleep duration, smoking, alcohol intake, history of steroid drug use, dyslipidaemia, and coronary heart disease, as well as all variables shown in the table.

**Table 5:** Interactions of risk factors for prediabetes among the elderly in rural areas.

Factor 1	Factor 2	OR (95% CI)^†^	RERI (95% CI)	AP (95% CI)	S (95% CI)
High WHR	Physical inactivity		6.30 (0.42, 12.18)	0.70 (0.50, 0.91)	4.84 (1.65, 17.85)
−	−	1.00			
+	−	1.96 (1.23, 3.12)			
−	+	1.68 (0.67, 4.23)			
+	+	8.94 (4.08, 19.57)			
High WHR	Overweight or obesity		2.92 (0.56, 5.29)	0.54 (0.28, 0.81)	3.02 (1.17, 7.46)
−	−	1.00			
+	−	2.00 (1.22, 3.27)			
−	+	1.45 (0.64, 3.28)			
+	+	5.37 (3.05, 9.44)			

†Adjusted for age, occupation, marital status, education, personal annual income, history of hyperglycaemia, sleep duration, family history of diabetes, smoking, alcohol intake, history of steroid drug use, health literacy on diabetes prevention and control, dietary patterns, hypertension, dyslipidaemia, and coronary heart disease, as well as the variables shown in the table. WHR: waist-to-hip ratio; RERI: relative excess risk due to interaction; AP: attributable proportion due to interaction; S: synergy index.

## Data Availability

The data analysed during this study are included in the article. The numerical data used to support the findings of this study are available from the corresponding author upon reasonable request.
